# Epstein Barr Virus Associated Lymphomas and Epithelia Cancers in Humans

**DOI:** 10.7150/jca.37282

**Published:** 2020-01-17

**Authors:** Richmond Ayee, Maame Ekua Oforiwaa Ofori, Edward Wright, Osbourne Quaye

**Affiliations:** 1 Department of Biochemistry, Cell and Molecular Biology, University of Ghana, Legon, Accra, Ghana; 2 West African Center for Cell Biology of Infectious Pathogens (WACCBIP), University of Ghana, Legon, Accra, Ghana; 3 Department of Biochemistry, University of Sussex, Brighton, U.K

**Keywords:** Epstein Barr virus, lymphomas, epithelial cancers, latency program

## Abstract

Epstein Barr virus (EBV) is a cosmopolitan oncogenic virus, infecting about 90% of the world's population and it is associated to tumors originating from both epithelia and hematopoietic cells. Transmission of the virus is mainly through oral secretions; however, transmission through organ transplantation and blood transfusion has been reported. In order to evade immune recognition, EBV establishes latent infection in B lymphocytes where it expresses limited sets of proteins called EBV transcription programs (ETPs), including six nuclear antigens (EBNAs), three latent membrane proteins (LMP), and untranslated RNA called EBV encoded RNA (EBER), shown to efficiently transform B cells into lymphoblastic cells. These programs undergo different patterns of expression which determine the occurrence of distinct types of latency in the pathogenesis of a particular tumor. Hematopoietic cell derived tumors include but not limited to Burkitt's lymphoma, Hodgkin lymphoma, post-transplant lymphoproliferative disorders, and natural killer (NK)/T cell lymphoma. EBV undergoes lytic infection in epithelia cells for amplification of the viral particle for transmission where it expresses lytic stage genes. However, for reasons yet to be unveiled, EBV switches from the expression of lytic stage genes to the expression of ETPs in epithelia cells. The expression of the ETPs lead to the transformation of epithelia cells into permanently proliferating cells, resulting in epithelia cell derived malignancies such as nasopharyngeal cancer, gastric cancer, and breast cancer. In this review, we have summarized the current updates on EBV associated epithelial and B cell-derived malignancies, and the role of EBV latency gene products in the pathogenesis of the cancers, and have suggested areas for future studies when considering therapeutic measures

## Introduction

Human gammaherpes virus 4 (HHV-4) or Epstein Barr virus (EBV) is a ubiquitous oncogenic virus belonging to the family *Herpesviridae* 1, 2, which is further classified into the subfamilies alpha, beta, and gammaherpes virus. EBV is a classical member of the subfamily *Gammaherpesvirinae* and among nine viruses that have been identified to solely infect humans 3, 4. The virus was first discovered and isolated in cells from African Burkitt's lymphoma by Epstein Barr and Achong in 1964 5, 6, and have been reported to establish latent asymptomatic infection in about 90% of the world's adult human population 7. Socioeconomic and developmental factors have been shown to influence the age at which primary infection can occur. For instance, in Sub-Saharan African countries where standard of living is poor, primary infection occurs in early childhood and most infected children seroconvert by the age of 3 years, whereas in developed or affluent countries, primary infection is delayed until late childhood or young adulthood 8. To establish primary infection, the virus is transmitted through oral route where it exhibits dual tropism by infecting two main physiological targets, epithelial cells and B lymphocytes 3. In addition to infecting the epithelia and B cells, the virus has also been shown to infect unnatural targets such as T lymphocytes and natural killer (NK) cells 9.

**Figure 1:**
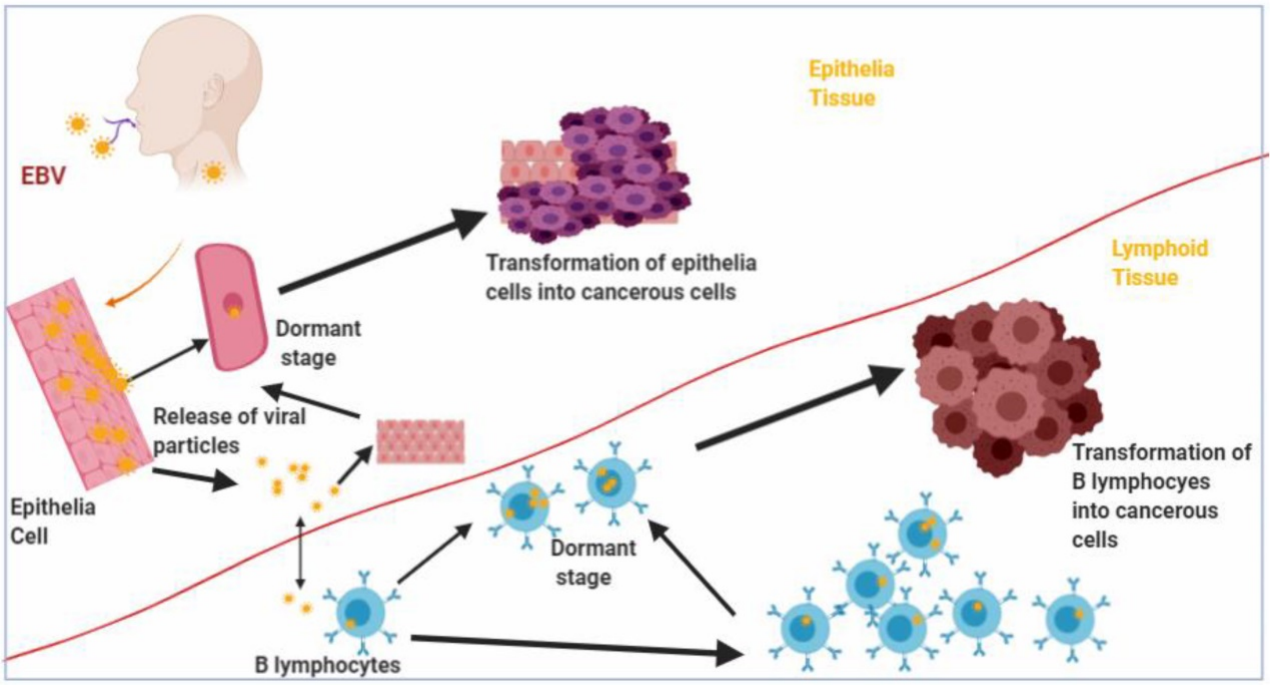
** Transformation of B lymphocytes and Epithelia cells into malignant cells by Epstein Barr virus (EBV)**. Epithelia and B lymphocytes are transformed by EBV into malignant cells as a result of expression of EBV latency gene products.

Lytic replication of the virus occurs in the epithelial cells, but the virus can establish latency by infecting B cells found in the pharyngeal lymphoid tissues of the Waldeyer's ring 7, 10. Upon entering the B cells, the viral genome either gets integrated into the host genome and persist as a provirus 11 or remain in the nucleus as a non-integrated circular episome and expresses restricted set of genes that drive latency and survival of the host cell 12, 13. The expression of the latency stage genes, known as latency programs, in the B cells lead to B cell-derived lymphomas as a result of the transformation of the cells into lymphoblastic lines (Figure 1). The virus can be reactivated from latency in the B cells by a mechanism that is yet to be elucidated. In immunocompetent individuals, viral titres are held in check by EBV specific cytotoxic T cells 14. Although EBV undergoes lytic replication in the epithelial cells, where lytic stage genes are expressed, the virus can switch to the expression of latency stage genes, and lead to the transformation of the epithelial cells into permanently proliferating cells and resulting in epithelial cell derived malignancies (Figure 1) 15.

In this review, we have summarized the current updates on EBV associated epithelial and B cell derived malignancies, and the role of EBV latency gene products in the pathogenesis of the cancers. In addition, we suggested areas that can be explored by researchers in future studies when considering therapeutic measures against these malignancies.

## Genome structure of EBV

The genome of EBV is a linear, double stranded DNA, approximately 172 Kbp with more than 85 protein coding genes (Open reading frames (ORF)) 16, 17. The ORFs encode proteins involved in regulation of DNA replication and gene expression, and maintenance of genome integrity in daughter cells 18. The nomenclatures of the ORFs were determined based on the *Bam* HI restriction fraction map in which the genes were in decreasing order according to their sizes 19. The protein coding genes are divided into lytic and latent genes which play structural and non-structural roles. The viral genome is further divided into long and short unique sequence domains which are separated by a series of 0.5 Kbp terminal direct repeats that are found at the end of each sequence domain 20. The terminal direct repeats increase the coding capacity of the genome and serve as a biomarker to determine the progenitor origin of EBV in the infected cells 17, 21.

## Classification and geographical distribution of EBV

EBV can be classified into two main genotypes, A and B, or types 1 and 2, based on sequence divergence in the nuclear proteins, Epstein Barr Nuclear Antigen (EBNA)-1, -2, -3A, -3B, and 3C 22. However, EBNA-2 is generally used for the classification because the protein has the least percentage of sequence homology between the two genotypes 23, 24. The two EBV genotypes occur worldwide but vary in geographical distribution; EBV type 1 is found globally but predominant in American, Chinese, European and South-East Asian (SEA) populations whereas type 2 is predominantly found in Africa 25. The two genotypes also vary in biological properties; EBV type 1 is more efficient in immortalizing B cells and the type 2 has a higher lytic ability 26, 27. The virus is further classified into seven different strains: China 1 (C1), China 2 (C2), China 3 (C3), Mediterranean with (Med+) or without (Med-) deletions, Alaskan (AL), and North Carolina, based on nucleotide polymorphism, 30 bp deletions, or signature amino acid changes in the LMP1 gene as compared to the sequence of a prototype EBV, B95-8 22, 28. The different strains show diversity in tumorigenic activity; a 30 bp deletion at the carboxyl terminal, for example, has a higher tumorigenic activity and poor immunogenicity compared to the undeleted variant 29.

## Biological activities of EBV-encoded latency gene products

Establishment of latent infection by EBV has been implicated in several malignancies 30 due to the expression of limited sets of latent proteins, shown to play various biological roles discussed in Table 1.

## Epstein Barr nuclear antigens (EBNAs)

**Figure 2:**
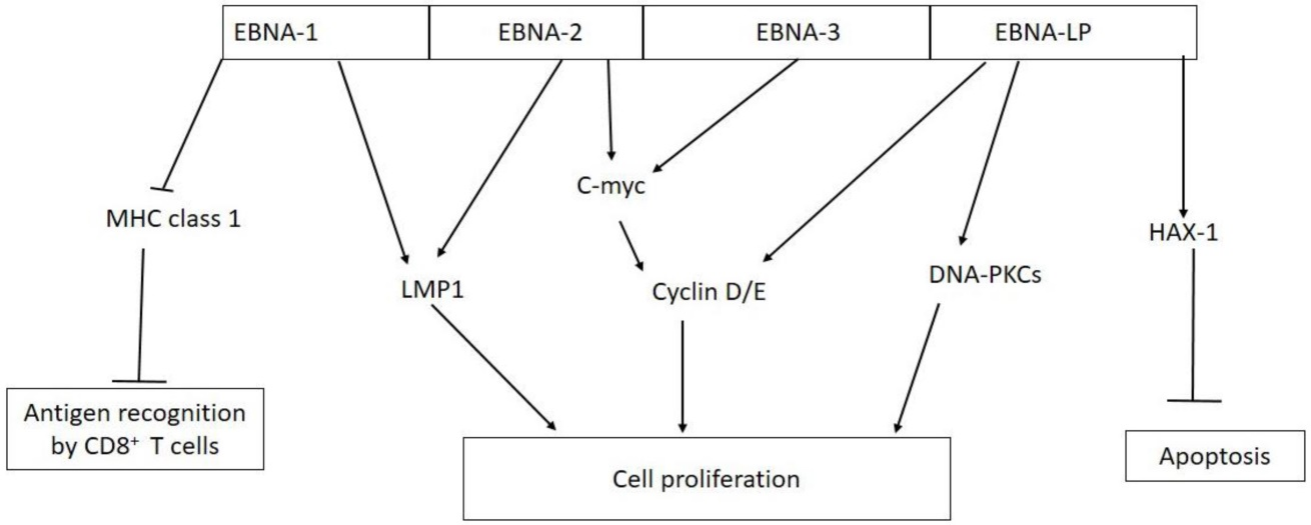
** Biological activities of EBV nuclear antigens (EBNAs) in tumorigenesis**. EBNA-1 inhibits antigen presentation by major histocompatibility complex (MHC) class I; EBNA-1 and EBNA-2 activate the expression of LMP-1; EBNA-2 and EBNA-3 interact with C-myc which constitutively activate cyclin D/E leading to unregulated cell proliferation; EBNA-LP can directly activate cyclin D/E and DNA-dependent protein kinase (DNA-PKCs) to promote cell proliferation; EBNA-LP promotes cell survival by interacting with antiapoptotic protein hematopoietic cell-specific protein 1 (HS-1)-associated protein X-1 (HAX-1).

Six EBNAs (1, 2, 3A, 3B, 3C and LP) are expressed during latent infection in host cells, and their biological functions have been reported by researchers worldwide 31, 32. Figure 2 shows the biological activities of EBV nuclear antigens (EBNA) in tumorigenesis. The expression of these antigens varies across the latency programs establish by EBV in infected cells. During latency III, all the six EBNAs are expressed, however in latency II and I, only one EBNA (EBNA-1) is expressed, but no EBNA protein is expressed during latency 0 8, 32. All the EBNAs are involved in regulation of transcription and also participate in transcriptional activation of viral LMP genes (*LMP*-1 and -2) in infected cells 33, 34.

EBNA-1 is a sequence specific DNA-binding protein that interacts with three unique palindromic sequence target sites (family of repeat elements (FR), dyad symmetry element (DS), and sequences found downstream Q promoter (Qp)), which are repeated multiple times on the viral genome 35. EBNA-1 binds to FR elements, which act as enhancer for viral C promoter, to direct the transcription of all the six EBNAs. Whereas engaging the DS elements by EBNA-1 leads to regulation of S-phase associated viral DNA replication, interaction with Qp down-regulates the transcription of Qp-driven EBNA-1 36, 37. EBNA-1 also ensures the segregation of the viral genome in daughter cells and up-regulates LMP promoter, sustaining cell survival or immortalization of infected cells 32. A more recent study has reported that EBNA-1 derived from nasopharyngeal cancer is required for the maintenance of EBV episome and DNA replication during latent infection 38. EBNA-1 protein is made up of N and C termini which are separated from each other by variable repeats of glycine-alanine sequences. The amino acid sequence repeats hide EBNA-1 from immune recognition by a cis-acting inhibitor of major histocompatibility complex (MHC) class I and preventing antigen presentation through ubiquitin- proteosome pathway 39, 40.

Unlike EBNA-1 which binds to DNA, EBNA-2 does not interact with DNA but engage cellular transcription factors such as CBF1/RBP-Jk, PU.1, and other proteins to up-regulate the expression of both viral and cellular genes 41. The cellular genes that are up-regulated by EBNA-2 include CD 23 and *c-myc* found in B cells. Activation of *c-myc* leads to expression of proteins such as D-type cyclins and cyclin E, which are associated with cell division 42. EBNA-2 also interacts with other transcription factors such as Cp, LMP-1, and -2 binding factors for up-regulation of viral genes that are responsible for immortalization of B cells 41, 43, 44.

EBNA-3 family of nuclear proteins consist of three large nuclear phosphoproteins, EBNA-3A, -3B, and -3C 44. All the EBNA-3 proteins share few sequence homologies at the N terminus region, with the conserved domain used to engage cellular transcription factors such as CBF1/RBP-Jk 45. By binding to CBF1/RBP-Jk, EBNA-3 acts as a repressor of EBNA-2-mediated transactivation of cellular and viral proteins 46. *In vitro* genetic studies have shown that only EBNA-3A and -3C are required for immortalization of EBV-infected cells 47, 48. Although EBNA-3 B is dispensable, a report has shown that the protein regulates B cell homing by altering the expression of chemokine receptor 4 (CXCR4) 49.

Epstein Barr nuclear antigen leader protein (EBNA-LP) also known as EBNA-5 is a nuclear phosphoprotein that is co-expressed with EBNA-2 upon EBV infection of target cells 50. The co-expression of EBNA-LP and EBNA-2 has been shown to enhance EBNA-2 transcriptional activation of both cellular and viral proteins 44, 51. EBNA-LP plays a key role in B cell transformation into a permanently proliferating lymphoblast based on the observation that cells expressing the mutant genes were less transformed as compared to cells with wild type EBNA-LP gene 52, 53. The leader protein interacts with cellular proteins, some of which are oncogenic and tumor suppressors (pRb, p53, p14ARF, and Fte1/S3a), cell cycle regulators (DNA-PKcs and HA95), and an anti-apoptotic protein (HAX-1) 54-56. EBNA-LP up-regulates the expression of thymus and activation regulated chemokine (TARC) gene, which has been proposed to play a key role in B cell transformation and survival 57, 58. Previous studies have reported that EBNA-LP stimulates EBNA-2 transactivation of viral proteins such as latent membrane proteins 1 and -2B (LMP-1 and -2B) in immortalized cells 51, 59, 60.

## Latent membrane protein (LMP)

**Figure 3:**
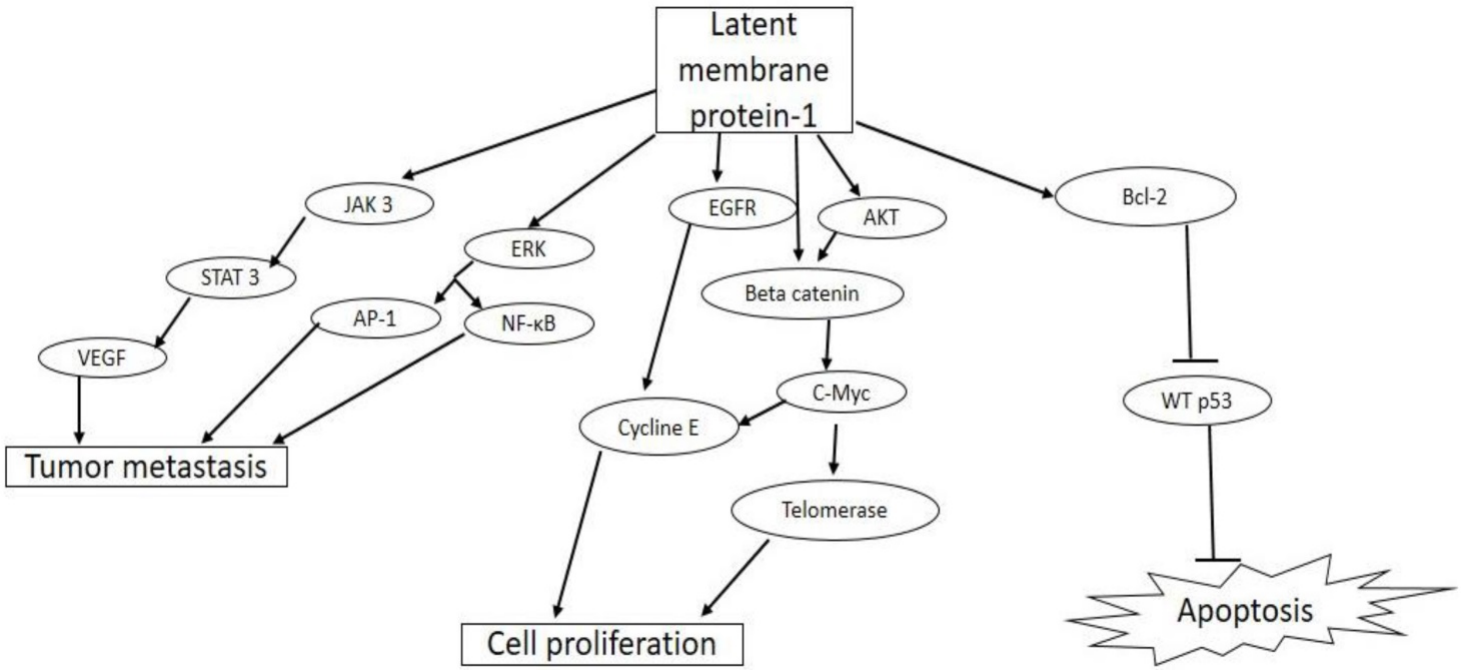
** Biological activities of latent membrane protein-1(LMP-1) in tumorigenesis.** EBV LMP-1 activates cellular pathways that lead to tumor invasiveness and metastasis, cell proliferation, and inhibition of apoptosis.

Three latent membrane proteins, LMP-1-2A, and -2B, are expressed by EBV during latency II and III in EBV infected cells 61. All the three proteins are expressed as cell surface membrane proteins and are required for survival and transformation of the infected cells into permanently proliferating cells (Figure 3).

LMP-1 gene is an essential oncogene, which is expressed as a constitutively active receptor in a majority of EBV associated tumor cells 62. LMP-1 is a 356-amino acid protein with a short cytoplasmic N-terminal domain, six transmembrane spanning domains, and a 200 amino acid long C-terminal cytoplasmic domain 63. The N-terminal domain tethers the LMP-1 on the surface of the plasma membrane, and oligomerization as well as self-aggregation of the protein is mediated by the six trans-membrane spanning domain. The C-terminal is the biologically active region of LMP-1, with two functional domains, namely, C-terminal activating regions (CTAR)-1 and -2, which are needed for efficient EBV-mediated B cell transformation 64. Previous reports have shown that these two functional domains interact with intracellular signaling proteins such as tumor necrosis factor (TNF) receptor-associated factor (TRAF)-1, -2, -3, -4, -5, and -6 found in tumor necrosis factor receptor (TNFR) signaling pathway in B cells and epithelia cells 65, 66. By interacting with these adaptor molecules, CTAR constitutively activates major signaling pathways such as the extracellular signal-regulated kinase (ERK), mitogen-activated protein kinases (MAPKs), PI3K/Akt, AP-1, Jun N-terminal protein kinase (JNK), the p38 and canonical and non-canonical NF-κB, and JAK/STAT pathways 67-69. The activation of these major signaling pathways results in the up-regulation of expression of antiapoptotic proteins such as A 20, Bcl-2, and down-regulation of tumor suppressor protein, p53, promoting cell growth and survival 70, 71. LMP-1 activates the Janus kinase 3 (JAK3)/Signal transducer and activator of transcription 3 (STAT3) pathway, which increases the expression of vascular endothelial growth factor (VEGF) and promotes tumor metastasis and invasiveness 72. LMP-1 also promotes proliferation of EBV infected cells through the activation of epidermal growth factor receptor (EGFR) and AKT/protein kinase B signaling pathways with downstream activation of cyclin E and telomerase, respectively 73. A more recent study has shown that the activation of AP-1, JAK/STAT, and NF-κB pathways by CTAR resulted in increased expression of programmed cell death protein 1 ligand (PD-L1), a key immune checkpoint suppressor in tumor immunology 74.

LMP-2 is made of two variants, LMP-2A and -2B, which are expressed during latency II and III in EBV infected cells 8. Structurally, both proteins have 12 trans-membrane domains and a 27 amino acid cytoplasmic C-terminal domain, however, LMP-2A has an additional 119 amino acid cytoplasmic N-terminal domain, which is not found in LMP-2B 75. The N-terminal cytoplasmic domain of LMP-2A contains immunoreceptor tyrosine-based activation motif (ITAM), which mediate B cell receptor (BCR)-like signaling cascade in EBV infected cells 76, 77. The BCR-like signaling, mediated by LMP-2A, provides strong survival signal which rescues BCR-negative cells from apoptosis 78, 79 and inhibit signaling that leads to lytic reactivation 80. LMP-2A has been shown to be essential for activation, proliferation, and survival of EBV infected B cells at early times, after which it is required for long term growth of B cells 81. Further study has shown that LMP-2A helps EBV infected cells to escape elimination by CD8^+^ T cells by down regulating (i) the expression of CD8^+^ T cells specific latent stage genes, especially EBNA-1, (ii) the expression of MHC class 1 proteins to prevent antigen presentation, and (iii) the expression of coactivatory receptor NKG2D to evade T cell recognition 82. The function of LMP-2B on the other hand, is not clearly known 81, but a previous report has shown that it interacts with LMP-2A to modulate the activities of the latter 75.

## EBV-microRNAs (EBV-miRNA)

MicroRNAs (miRNA) are small non-coding RNA molecules, usually 18-24 nucleotides in length, which transcriptionally down-regulate expression of complementary mRNAs 83. miRNAs have been identified in different organisms such as mammals, plants, algae and worms, and EBV was one of the first viruses that was identified to express these small non-coding RNA molecules 84, 85. The virus has been found to express 44 miRNAs in all forms of latency and tumor tissues, regulating both cellular and viral genes 86. The 44 miRNAs are produced from two miRNA coding regions: the BamHI fragment H rightward open reading frame 1 (BHRF1) region which encoded 4 miRNAs, and the BamHI A rightward transcript (BART) region which encodes the remaining 40 miRNAs 87. Since EBV miRNAs are detected in EBV associated tumors, it is thought that they play important roles in the pathobiology of the life cycle of EBV and tumors associated to EBV 88. miRNAs help the virus to escape immune surveillance by down-regulating the expression of both immunogenic viral antigens and host immune proteins 83. For instance, Lung and colleagues in 2009 reported that BART 22, encoded by the BART miRNA region, down-regulated the expression of LMP-2A, an immunogenic viral protein, and enhanced NPC tumorigenesis 89. Impotin 7 (IPO7), which is a cellular receptor responsible for transporting transcription factors into the nucleus and plays a key role in innate immunity, has been shown to be inhibited by EBV miR-BART3 and miR-BART16 90, 91, and miR-BART2-5p protects EBV-infected cells from immune recognition by natural killer (NK) cells through the inhibition of major histocompatibility complex (MHC) class I chain-related molecule B (MICB) 92, 93. Other studies have also demonstrated that EBV miRNA BART 16 disrupt Type I IFN signaling pathway by down-regulating the expression of CREB-binding protein, a transcriptional activator of IFN, and hence suppressing host immune response against the virus 94, and EBV produces BART6-3p which interacts with miR 97, a cellular microRNA, to synergistically suppress the expression of interleukin-6 receptor (IL-6R), which is a receptor for several antiviral cytokines such as IFN-α, IL-12 and IL-27 95, to promote tumor progression in both epithelia cancers and lymphomas.

Aside targeting cellular cytokines, EBV miRNAs inhibit the destruction of B cell and epithelia cell derived cancers by down-regulating expression of tumor suppressor genes; miR-BHRF1-2 has been reported to silence the expression of PRDM1/Blimp1which is a tumor suppressor in B cells, hence promoting lymphogenesis 96. The virus also targets the mitogen-activated protein kinase (MAPK) signaling pathway, which suppresses tumor formation by producing BART 22 and shown to inhibit MAP kinase kinase kinase 5 (MAP3K5) expression 97.

## Detection of EBV

EBV is implicated in several malignancies ranging from epithelial and lymphoid origins. Biochemical, serological, and molecular methods to detect EBV have increasingly become necessary in the diagnosis and monitoring of patients with EBV-associated diseases 98. Serological assays, that are used to identify the virus in infected individuals, involve the detection of antibodies against EBV specific antigens such as viral coat antigen (VCA), EBNA-1, early antigen (EA) and viral nucleic acids 99-101. Immunohistochemistry, which involves the staining of key EBV latency proteins such as LMP-1, LMP-2A, EBNA-1, and -2 in tumor biopsies, is used to confirm the presence of the virus and distinguish between EBV-associated and non EBV-associated tumors 102. Epstein Barr encoded RNA (EBER) *in situ* hybridization (EISH) assay is considered as the gold standard for the detection and diagnosis of EBV infection 103. The EISH technique employ the use of nucleic acid probes, either label or unlabeled, which can hybridize with EBER on paraffin sections of EBV infected tissues 104. EBER is a small non-coding and non-polyadenylated RNA which is highly expressed in all EBV latently infected cells, irrespective of cell phenotype. Detection and quantification of EBV nucleic acids in body fluids and tissues using polymerase chain reaction (PCR) have also been exploited for diagnosing and monitoring of EBV-associated diseases 105.

## Malignancies caused by EBV infection

**Figure 4:**
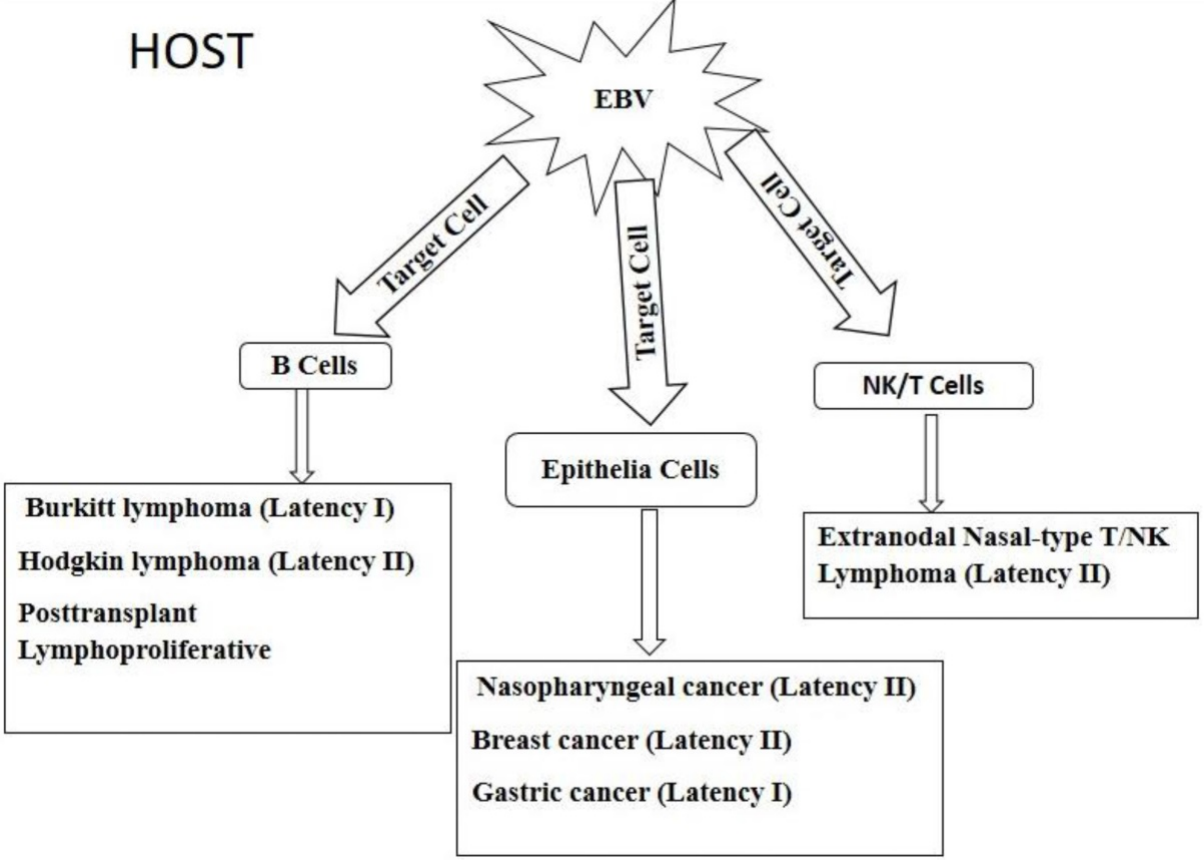
Diagrammatic view of EBV associated epithelia and hematopoietic cell derived malignancies.

EBV is linked to the pathogenesis of lymphomas such as Burkitt's lymphoma (BL), Hodgkin lymphoma (HL) and post-transplant lympho-proliferative disorder (PTLD). Epithelial malignancies that are associated with EBV in epithelia cells include nasopharyngeal cancer (NPC), gastric cancer (GC) and breast cancer (BC) 34. In rare cases, EBV has been shown to infect NK and T cells; causing extranodal nasal-type NK/T cell lymphoma 106. The different malignancies are shown in Figure 4. Upon entering the host, EBV has tropism for two main targets, namely B cell and epithelia cells as well as unnatural targets such as NK/T cells. The virus in the various target cells expresses different patterns of latency genes and these determines the type of cancer that would be developed. B cell derived lymphomas include Burkitt lymphoma, Hodgkin lymphoma, and posttransplant lymphoproliferative disorders which are caused by the expression of latency I, II, and III, respectively, gene products. Infection of epithelia cells by EBV results in cancers such as nasopharyngeal, breast, and gastric cancers, caused by the expression of latency II, II, I, respectively, gene products. The virus can also invade unusual targets such as natural killer (NK) cells and T lymphocytes to cause extranodal nasal-type T/NK lymphoma due to the expression of latency II gene products. Full details of each tumor are further described below.

## Burkitt's lymphoma (BL)

Burkitt's lymphoma (BL) is a highly aggressive non-Hodgkin B cell neoplasm and is the world's commonest pediatric cancer; endemic in Sub-Saharan Africa 107. The pathogenesis of BL has been linked to EBV infection as a result of the isolation of the virus from cultured BL cell lines in 1964 5. The virus has since been implicated in 95% of BL cases from high risk regions and less than 30% from low risk regions 108. BL occurs in children living in areas that are both holo-endemic and hyper-endemic for malaria and suggesting that *Plasmodium falciparum* plays a role in the etiology of the cancer 109, 110. BL is classified into three different forms based on clinical observations and disease epidemiology 111. Endemic BL (eBL) has an annual incidence of about 5-10 cases per 100 000 and contributes 50% of all pediatric cancers in malaria endemic regions such as Equatorial Africa and Papua New Guinea, where EBV is found in about 95% of all diagnosed cases 112, 113. Common sites of tumor occurrence of eBL are the jaws and abdomen 114. Sporadic Burkitt's lymphoma (sBL), on the other hand, has a wide global distribution, but is at a much lower frequency and mostly diagnose in both children and young adult. In US and Western Europe, sBL is responsible for 30-50% of all pediatric neoplasms and less than 1% in adult 115. EBV is rarely linked to sBL cases diagnosed in the western world, however in other places like North East Brazil, the frequency of EBV in sBL exceeds 80% 7, 116. In contrast, immunodeficiency-associated Burkitt's lymphoma (iBL) has been found in HIV carriers who develop the lymphoma before progressing to AIDS. The incidence of iBL is 10 to 100-fold higher than the sporadic form of the disease, and about 30-40% of iBL are positive for EBV 117. Immune compromise, which occurs as a result of HIV infection, accounts for reactivation of EBV from latently infected B cells and eventually causing rapid progression to iBL 118. CD4+ T cells prime CD8+ T cells against circulating EBV in immunocompetent individuals, and thereby bringing the virus under immune surveillance. However, in HIV infected individuals the CD4+ T cell count is drastically reduced, and therefore reduces the priming of the EBV specific CD8+ T cells in these individuals 119, 120.

The expression of EBV genes in BL is strictly latent and restricted to the type I latency default programs. Only EBNA-1 and EBER are expressed in EBV positive cells in BL tumors 121. Whiles some studies have reported the detection of LMP-1 and EBNA-2 in rare cases of eBL and LMP-1 in many sBL cancers, these detections were not observed in all occurrences 122-124. Even though the role of EBV in the pathogenesis of BL is still unclear, evidence have shown that the expression of EBNA-1 in BL cell lines promotes cell proliferation by inhibiting apoptosis 125. Recent reports suggest that EBNA-1 inhibits apoptosis in BL cell lines by interacting with host proteins such as survivin, an anti-apoptotic protein and p53-regulator USP7 38, 126.

All BL tumors, regardless of variants or EBV status, are morphologically and immunophenotypically identical and are related by sharing a general gene expression patterns which resemble that of centroblasts 127, 128. The BLs, in general, undergo chromosomal translocation of *c-myc* proto-oncogene on chromosome 8 or on chromosome 14 to one of the three immunoglobin loci, notably the heavy chain immunoglobin (Ig) 129. This brings the *c-myc* under the control of highly active Ig gene promoter leading to constitutive expression of *c-myc* proteins at high levels in BL cells. The expression results in uncontrolled cell growth in BL 130.

## Hodgkin lymphoma (HL)

Hodgkin lymphoma (HL) is a lymphoid neoplasm which originates from B cell and characterized by the presence of few multinucleated giant cells known as Hodgkin/Reed Sternberg (HRS) cells, which are surrounded by a non-neoplastic inflammatory infiltrate 131. The incidence of HL varies depending on geographical distribution, sex, ethnicity, and socioeconomic status; the incidence is higher in developed countries compared to under-developed ones. 132. There is however lower mortality due to HL in developed countries compared to the less developed ones; an observation that has been ascribed to improved health facilities in the developed countries 133. About 30-40% of HL cases in North America and Europe were reported to be EBV positive, while in other parts of the world such as Latin America, Asia and Africa, EBV was found in almost 100% of all HL cases 134. Despite the association of EBV to HL cases, the role of EBV in the pathogenesis of this malignancy remains unknown 135.

There are two variants of HL based on clinical and morphological differences 136. The first variant, known as nodular lymphocyte predominant Hodgkin lymphoma (NLPHL), is rarely EBV associated 137, hence would not be discussed in this review. The second variant, known as classical Hodgkin lymphoma (CHL), has been reported to account for about 95% of all HL cases and is mostly linked to EBV 138. Association between EBV and CHL has been suggested due to elevated antibody titres against EBV latent antigens, which proceeded the development of the lymphoma after several years of infection 139. Detection of EBV latent stage genes and RNA from biopsies of CHL patients was further used to confirm the association between the disease and EBV 140. CHL can further be divided into four histological subtypes, namely, mixed cellularity (MC), nodular sclerosis (NS), lymphocyte-rich (LR), and lymphocyte depleted (LD) 138. About 96% of all MC cases are EBV associated, whereas NS is less frequently linked to EBV infection 141. On the other hand, LR and LD are rarely associated with EBV 142.

## Post-transplant lymphoproliferative disorder (PTLD)

Post-transplant lymphoproliferative disorder (PTLD) is a type of malignancy with life-threatening complications in transplant recipients of both solid organ and hematopoietic stem cell allografts 143. The lymphoma occurs as a result of increase proliferation of B cells after transplant, and it is mostly EBV driven 144. The genome of the virus has been found in more than 90% of B cells from PTLD patients, either after primary infection, or reactivation of EBV from latently infected cells after treatment with immunosuppressant to avoid allograft rejection 145. The use of immune suppressive drugs after organ transplant reduces the number and function of T cell to influence the success of the transplant. The use of the immunosuppressant leads to depletion of EBV-specific T cells and disruption of the balance between the immune system and latent virus, and consequently, there is reactivation of EBV from latency 146. The impairment of EBV-specific T cell mediated immune surveillance results in uncontrolled lymphoproliferative blast, which eventually causes PTLD in transplant recipients 147, 148.

The prevalence of EBV- associated PTLD ranges from 1-20%, with incidence varying according to the type of allograft, age, and pretransplant EBV-serostatus of transplant recipient. PTLD cases are highest (32%) in small intestine transplant recipients, and 3-12% incidence is observed in heart, lung, liver and pancreas transplant recipients. The lowest incidence (1-2%) is reported in renal transplant recipients 146, 149. At the ages of 5, 18, and 40, about 50, 20, 5-10% of the general population, respectively, have not been exposed to EBV, hence are seronegative. These groups of people develop primary infection after receiving transplant from a donor who is EBV seropositive, predisposing them to PTLD 150, 151. The overall incidence of PTLD is found to be higher in children than in adults due to increased pretransplant EBV seronegative status among children 146.

## Nasopharyngeal cancer (NPC)

Nasopharyngeal cancer (NPC) is a malignant tumor of epithelia squamous cell which arises from the lateral wall of nasopharynx, notably, fossa of Rosenmüller and superior posterior wall 152. Globally, the disease shows remarkable variation in ethnic and geographical distribution, with about 80% of a total of 65,000 new cases that are reported worldwide, occurring in Southern China and Southeast Asia. 153. The annual incidence of NPC in Southern China varies between 20-30 per 100,000 person-year among Cantonese living in Hong Kong and Guangdong province, while the incidence in other parts of the world, such as Europe and the United State, are below 1 per 100,000 persons/year 154, 155. In Africa, specifically, Northern Africa, the incidence of NPC varies between 5 to 7 per 100 000 persons/year 156. Based on histology, the World Health Organization (WHO) classified NPC into types I, II, and III, which are known as keratinizing squamous cell, differentiated non-keratinizing, and undifferentiated non-keratinizing carcinomas, respectively 157. An alternatively simpler system of classification has been proposed which groups NPC into two histological variants, namely squamous cell carcinomas (SCCs) and undifferentiated carcinomas of the nasopharyngeal type (UCNT) 158. In a non-endemic region like North America, about 63% of all NPC cases are UCNT, while in Southern China, about 95% of all the cases are UCNT 159.

EBV, which is the main cause of UCNT, has been classified as group 1 carcinogenic agent by the International Agency for Research and Cancer (IARC) 22. The viral infection in NPC epithelia cells is clonal in origin; developing from clonal proliferation of single EBV infected epithelia cell. NPC tumors express three EBV latency type II gene products (LMP-1, LMP-2, EBNA-1), which are found in EBV implicated epithelial cancers, in addition to the expression of viral encoded small RNAs such as EBER and microRNAs (miRNAs) 22, 100, 160. Of all EBV latency gene products, LMP-1 has been found in about two-thirds of NPC cases, indicating the key role this protein plays in tumorigenesis.

As previously mentioned, the infection of epithelia cells by EBV is mainly lytic, and hence default lytic programs are expressed. However, the switch to latency default programs during epithelia infection by EBV represents a key step in the pathogenesis of NPC, although some studies have reported the involvement of both lytic and latent stages EBV genes in transformation of epithelia cells, the role of lytic genes remains unclear 15, 161, 162.

## EBV-associated gastric cancer (EBVaGC)

Gastric cancer (GC) is the third leading cause of cancer-related mortality globally, with a worldwide annual incidence of over 950 000 cases. EBV-associated gastric cancer (EBVaGC) accounts for about 10% of all GC reported cases 163, 164. The incidence of EBVaGC shows variation in geographical distribution across the globe, with a pooled estimate in Europe, Asia, and North and South America, being 9.2%, 8.3% and 9.9%, respectively (Huang* et al.*, 2014), whereas in Africa, a country like Zambia has a frequency of 23% 165. EBVaGC is classified into three histological subtypes namely: lymphoepithelioma-like carcinoma (LELC)-type, conventional type adenocarcinoma (CA)-type, and carcinoma Crohn's disease- like lymphoid reaction (CLR)-type 166. The LELC-type is a poorly differentiated carcinoma with dense infiltration of lymphocytes, similar to that of NPC. More than 80% of EBVaGCs show LELC-type morphology 167. CA-type on the other hand, morphologically resembles EBV negative GCs by infiltration of variable lymphocytes with prominent desmoplasia in the absence of lymphoid follicles 168. CLR-type is characterized by the presence of three or more lymphoid follicle with active germinal centers located at the advancing edge of the neoplasm 168.

One of the unique features which distinguishes EBVaGC from non-EBVaGC is the monoclonal proliferation of gastric epithelia cells latently infected with EBV and suggesting the presence of the virus in the early stages of tumorigenesis 169. In contrast to EBV-negative GCs, which mainly occur in the antrum as the predominant pathological site, EBVaGC occurs predominantly in the proximal stomach including the cardia, fundus and body 168. At early stages of tumorigenesis, EBVaGC forms a well-defined nodular ulcer in the submucosa with less fibrosis as compared to non-EBVaGCs, and this pathological feature is essential for endoscopic submucosal resection of the tumor 170.

The genome of EBV, mainly Latency I default programs (EBER, EBNA-1, and BART), was found in the GC cells and adjacent dysplastic epithelium cells, but could not be found in surrounding lymphocytes, stromal cells, normal mucosa, and intestinal metaplasia, and therefore suggests a key role of EBV in the pathogenesis of GC 171.

## Extranodal nasal-type NK/T-cell lymphoma

Extranodal nasal-type NK/T lymphomas are infrequent tumors that are regularly associated with EBV infection 172. The tumor is characterized by extensive angio-invasion and necrosis in the upper aerodigestive tract, including the nasal cavity, nasopharynx, paranasal sinus and the palate 173. The disease shows remarkable variation in geographical and sex distribution, with the highest incidence occurring in East Asia, Mexico, and South/Central America 174. The lymphoma has been shown to be predominant among men than woman, with 2-3:1 ratio of male to female, and the mean age at diagnosis being 50 years 175, 176.

EBV expresses type II latency programs (EBNA-1, LMP-1 and -2) in the extranodal NK/T-cell lymphomas, and the genome of the virus exists as a clonal episome in the infected cells 177. The expression of these genes has been shown to constitutively activate several signaling pathways including JAK/STAT and NF-κB, to promote cell growth and survival 178. LMP-1 expression in extranodal NK/T-cell lymphomas has also been reported to promote tumor immune escape through the up-regulation of programmed cell death receptor 1 (PD-1) and PD ligand 1, which are both important immune checkpoint molecules in tumor immunology 179.

Genome wide analysis have reported chromosomal abnormalities, notably, deletions in 6q21 in extranodal NK/T-cell lymphomas 180, which leads to the loss of expression of many tumor suppressor genes such as ATG5, PRDM1, FOXO3, AIM1, and HACE 1. For instance, the loss of FOXO3 and HACE1 in EBV-positive NK/T-cell lymphomas result in apoptosis resistance through the prevention of BIM and PUMA induction, and the suppression of TNF-driven NF-κB activation, respectively 181.

## Breast cancer

Breast cancer is the most frequently diagnosed female malignancy globally and accounts for about 25% of incident cancer cases among women 182. In underdeveloped regions such as sub-Saharan Africa, frequency of breast cancer is relatively lower, characterized by aggressive course and targets female of younger age compared to the Western World 183.

While genetic factors, such as inheritance of breast cancer associated gene 1 and 2 (*BRCA1/2*) and human epidermal growth factor receptor 2 (*HER2*), in addition to non-genetic factors, have been shown to influence the development of breast cancer, the etiology remains unknown 184. Viral agents including EBV, mouse mammary virus (MMV), human papilloma virus (HPV), and cytomegalovirus have been implicated in breast cancer cases 185-187. Association between EBV and breast cancer have been reported from different parts of the world including Asia 188, Africa 189, and Europe 190, with a prevalence of 30-50%. Subsequently, EBV latency II gene products such as LMP-1, -2, EBNA-, and EBER have been detected in breast cancer cells 102, 191, 192, and a meta-epidemiological study have also shown that EBV infection was highly associated with the risk of breast cancer development 193.

In keeping with the association between EBV and breast cancer, a proposed potential mechanism for the transformation of mammary epithelia cells (MEC) suggests that EBV infects cells that express CD21 as cell surface receptor, leading to tumorigenesis that occurs through LMP-1 mediated activation of c-MET signaling pathway 194.

## Summary and future studies

Epstein Barr virus is an oncogenic virus with global distribution and infecting about 90% of the world's population. Humans are the only natural host of EBV, and the virus is transmitted by ingesting infected saliva. In the host, the virus has tropism for two main cells, namely epithelia cells and B lymphocytes, although unnatural targets such as natural killer cells and T lymphocytes have been shown to contain the virus. The presence of EBV in B cells has been linked to lymphomas, which include Burkitt's lymphoma, Hodgkin lymphoma, and posttransplant lymphoproliferative disorders. On the other hand, infection of epithelia cells is implicated in the pathogenesis of epithelia cell derived malignancies such as nasopharyngeal cancer; EBV associated gastric cancer, and breast cancer. The pathogenesis of both B cell and epithelia cell derived neoplasms has been due to the expression of EBV transcription programs or latency stage genes. The programs are divided into latency I (EBNA-1, EBER, and BARF0), latency II (EBNA-1, EBER, LMP-1, and -2), and latency III (EBER, EBNA-1,-2,-3,-4,-5, and LMP-1 and -2A), and their expression varies depending on the differentiation, type, and activation status of the target cell. Latency I programs are implicated in Burkitt's lymphoma and gastric cancer, whereas the expression of latency II default programs is linked to nasopharyngeal cancer, Hodgkin lymphoma, and NK/T-cell lymphomas. The pathogenesis of posttransplant lymphoproliferative disorders has however been associated with the expression of latency III default programs. Tumorigenesis occurs by (1) upregulating the expression of antiapoptotic genes, (2) constitutive activation of major intracellular signaling pathways responsible for cell growth and survival, and (3) creation of tumor microenvironment for malignant cells to escape immune recognition.

EBV have been linked to the pathogenesis of different malignancies, but the mechanism underlying the pathogenesis has not been fully elucidated. Host and environmental factors are thought to play key roles in the malignancies, making it difficult to clearly map out the mechanism involved in the pathogenesis of EBV-associated cancers. With current progress in genomic research, future studies should focus on how host genes and environmental factors interact with viral genes, and how the interaction influences the pathogenesis of EBV-associated malignancies. Due to overlapping symptoms, many EBV implicated cancers are only diagnosed at the advance stage of the disease. Genome-wide association studies and whole genome sequencing of large population of patients are therefore recommended to identify polymorphism in genes which predisposes individuals to the above mentioned EBV-associated cancers. Results from these studies will help identify high risk-populations for further prognostic evolution and increase the chances of detecting the cancers at early stages of development. Future studies must be done on anticancer drugs that selectively regulate the expression of EBV oncogenes involved in antiapoptosis, cell proliferation and invasion. Finally, selective inhibition of various signaling pathways activated by EBV proteins, offers promising anticancer therapy. All the proposed recommended studies will help understand the disease mechanism, and ultimately lead to the development of therapeutic agents that will help slow down disease progression and increase survival.

## Figures and Tables

**Table 1:** Biological activities of Epstein Barr virus latency stage gene products and associated cancers

EBV latency protein	Type of latency	Biological activity	Associated cancers^d^
**EBNA-1^a^**	Latency I, II, III	Segregation of viral genome in progenies, DNA replication, inhibition of MHC class I, enhances p53 degradation	Burkitt lymphoma, Gastric cancer, Breast cancer
**EBNA-2**	Latency III	Upregulation of host and viral proteins (transactivation), facilitate B cell immortalization	Posttransplant lymphoproliferative disorder
**EBNA-3**	Latency III	Transcription transactivation of both host and viral proteins, immortalization of B cell	Posttransplant lymphoproliferative disorder
**EBNA-LP^b^**	Latency III	Transactivation of EBNA-2 to inactivate tumor suppressors, essential for immortalization of B cells	Posttransplant lymphoproliferative disorder
**LMP-1/2^c^**	Latency II/III	B cell survival, upregulation of antiapoptotic proteins, mimics CD 40 ligand associated signaling, constitutively activate growth and cell survival promoting signaling pathways	Hodgkin lymphoma, Nasopharyngeal cancer, Posttransplant lymphoproliferative disorder, T/NK cell lymphoma, Breast cancer
**EBV-Micro RNAs**	Latency I, II, III	Target host mRNAs involved in apoptosis, proliferation and transformation. Suppress antigen presentation and activation of immune cells	Gastric cancer, T/NK cell lymphoma, nasopharyngeal cancer

**^a^** EBNA-1 is expressed and detected in all EBV associated malignancies. **^b^** EBNA-LP is also known as EBNA-5. **^c^** LMP-1/2 are both involved in epithelia and B cell tumors, however, LMP 2 is frequently detected in a majority of all tumors as compared to LMP-1. **^d^**The associated tumors are not only limited to the ones discussed in this review.

## Abbreviations

BC: Breast cancer
BL: Burkitt's lymphoma
EA: Early Antigen
EBER: Epstein Barr encoded RNA
EBNA: Epstein Barr nuclear antigen
EBV: Epstein Barr Virus
EBVaGC: Epstein Barr associated gastric cancer
GC: Gastric cancer
HHV-4: Human gamma herpes virus 4
HL: Hodgkin lymphoma
IHC: Immunohistochemistry
LMP: Latent membrane protein
NPC: Nasopharyngeal cancer
ORF: Open reading frame
PBMC: Peripheral blood mononuclear cells
PTLD: Post-transplant lymphoproliferative disorder
VCA: Viral coat antigen
